# Low-dose IL-2 enhances the generation of IL-10-producing immunoregulatory B cells

**DOI:** 10.1038/s41467-023-37424-w

**Published:** 2023-04-12

**Authors:** Akimichi Inaba, Zewen Kelvin Tuong, Tian X. Zhao, Andrew P. Stewart, Rebeccah Mathews, Lucy Truman, Rouchelle Sriranjan, Jane Kennet, Kourosh Saeb-Parsy, Linda Wicker, Frank Waldron-Lynch, Joseph Cheriyan, John A. Todd, Ziad Mallat, Menna R. Clatworthy

**Affiliations:** 1 Molecular Immunity Unit, University of Cambridge Department of Medicine, Cambridge, UK; 2 Cellular Genetics, Wellcome Sanger Institute, Hinxton, UK; 3 Department of Medicine, Division of Cardiovascular Medicine, University of Cambridge, Cambridge, UK; 4 Ear, Nose Throat Department, West Suffolk Hospital, Bury St Edmunds, UK; 5 Division of Experimental Medicine and Immunotherapeutics, Department of Medicine, University of Cambridge, Cambridge, UK; 6 Wellcome-MRC Institute of Metabolic Science-Metabolic Research Laboratories and Medical Research Council Metabolic Diseases Unit, University of Cambridge, Cambridge, UK; 7 Department of Surgery, University of Cambridge, Cambridge, UK; 8 National Institute for Health Research Cambridge Biomedical Research Centre, Cambridge, UK; 9 Medical Sciences Division, University of Oxford, Oxford, UK; 10 Novartis Institutes for BioMedical Research, Autoimmunity Transplantation Inflammation, Basel, Switzerland; 11 Wellcome Centre for Human Genetics, Nuffield Department of Medicine, University of Oxford, Oxford, UK; 12 Universite de Paris and INSERM, Paris, France

**Keywords:** Autoimmunity, Interleukins, Immunosuppression, Plasma cells, Translational immunology

## Abstract

Dysfunction of interleukin-10 producing regulatory B cells has been associated with the pathogenesis of autoimmune diseases, but whether regulatory B cells can be therapeutically induced in humans is currently unknown. Here we demonstrate that a subset of activated B cells expresses CD25, and the addition of low-dose recombinant IL-2 to in vitro stimulated peripheral blood and splenic human B cells augments IL-10 secretion. Administration of low dose IL-2, aldesleukin, to patients increases IL-10-producing B cells. Single-cell RNA sequencing of circulating immune cells isolated from low dose IL2-treated patients reveals an increase in plasmablast and plasma cell populations that are enriched for a regulatory B cell gene signature. The transcriptional repressor *BACH2* is significantly down-regulated in plasma cells from IL-2-treated patients, BACH2 binds to the IL-10 gene promoter, and *Bach2* depletion or genetic deficiency increases B cell IL-10, implicating BACH2 suppression as an important mechanism by which IL-2 may promote an immunoregulatory phenotype in B cells.

## Abstract (web summary)

The dysfunction of IL-10 secreting regulatory B cells has been linked to the pathogenesis of autoimmune disease. Here the authors show that low dose IL-2 therapy can enhance IL-10 production in regulatory B cell populations via the modulation of BACH2.

## Introduction

The capacity of the immune system to regulate its activation is critical for the prevention of pathogenic responses against self or commensal antigens. Central to this capability are subsets of cells that inhibit immune responses and mediate their effects by producing regulatory cytokines such as interleukin (IL)−10, or via contact-dependent inhibition. The best characterised immunomodulatory cells are regulatory T cell (Treg) populations, which are dependent on IL-2 for their maintenance and function. The IL-2 receptor has three subunits, CD132, the common γ-chain that is widely expressed and utilized by a number of other cytokine receptors, CD122 (β-chain) and CD25 (α-chain). Expression of CD25, in the presence of CD122 and CD132, generates a high affinity receptor and constitutive expression of CD25 is characteristic of Tregs. Indeed, administration of low dose (LD) IL-2 has been used as a therapeutic strategy to preferentially induce Treg expansion, without stimulating effector T cells, in a number of immune disorders including alopecia areata^1^, chronic graft-versus-host disease^2^, hepatitis-induced vasculitis^3^, systemic lupus erythematosus^4^, and type 1 diabetes (T1D)^5,6^, in order to limit harmful immune responses. In contrast, the effect of IL-2 treatment on the B cell compartment has received little attention to date.

There is an increasing appreciation that B cells also have the capacity to regulate immune responses, including via IL-10 production. In mouse models, the transfer of regulatory B cells (Bregs) can inhibit a number of autoimmune diseases including arthritis^7^, colitis^8^ and lupus^9^. Additionally Bregs have been shown to be important in inducing tolerance in transplantation^10^. Therefore, understanding factors which promote the development of Bregs in humans is of therapeutic interest. Studies of murine B cells in vivo and of murine and human peripheral B cells in vitro suggest that toll-like receptor (TLR)-ligation and T cell-derived signals, including CD40 ligand (CD40L) and IL-21, promote the generation of IL-10-producing Bregs^11^, but such strategies would be challenging to harness in patients.

In this work, since activated T cells are also a major source of IL-2 which is required for the generation and maintenance of Tregs, we asked whether IL-2 might also promote a regulatory phenotype in B cells, and if the administration of LD IL-2 could be used to expand IL-10-producing Bregs cells in humans in vivo. Indeed, we show that low dose IL-2 increases IL-10-producing B cells in-vivo by its influence on the transcriptional repressor BACH2.

## Results and discussion

### IL-2 receptor on the surface of Bregs

**Fig. 1:**
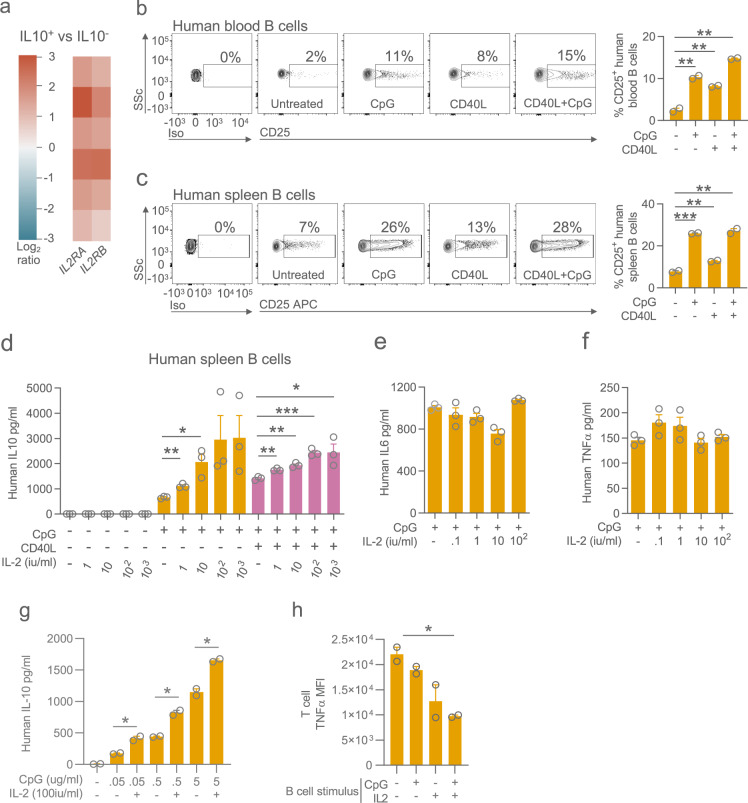
Activated B cells express CD25 and the addition of IL-2 increases IL-10 production by in vitro and vivo. **a** Heatmap showing *IL2RA and IL2RB* transcript expression in IL-10-positive human B cells relative to IL-10-negative B cells in *n* = 6 subjects^30^. **b** Flow cytometry plots and quantification of surface expression of CD25 (IL-2Rα) on human blood (**b**) and splenic (**c**) B cells. Gated on live single CD19^+^ events. All bar graphs in figures show mean and error bars representing standard error of mean (SEM). Representative of three experimental replicates. **d** Quantification of IL-10 in culture supernatants from human splenic B cells stimulated with IL-2, CpG and/or CD40L. Quantification of IL-6 (**e**) and TNFα (**f**) in culture supernatant from negatively-isolated human splenic B cells stimulated with IL-2 and CpG. Graphs show mean and SEM of triplicates and are representative of three experimental repeats. **g** Quantification of IL-10 in culture supernatants from negatively isolated human blood B cells stimulated with IL-2, and lower concentrations of CpG. **h** CD4 T cell TNFα MFI following co-culture with CpG-stimulated B cells generated in the presence or absence of IL-2. Graphs show mean and SEM of duplicates and are representative of three experimental repeats. *p*-values generated using an unpaired two-tailed parametric *t*-test. *p < 0.05, **p < 0.01, ***p < 0.001. Source data are provided as a Source Data file.

First, we investigated whether IL-10-producing B cells expressed CD25, the subunit generating a high affinity IL2R, in an analogous way to Tregs. We observed significantly higher CD25 transcripts in IL-10-positive human B cells compared with IL-10-negative B cells (Figs. 1a, S1a). In human peripheral blood, basal surface CD25 expression was detectable in only 2% of B cells (Fig. 1b). However, following activation in vitro with the TLR9 ligand CpG or with CD40L, we observed a six-fold increase in the proportion of CD25-positive cells (*p* < 0.001, Fig. 1b). Since B cell activation takes place in secondary lymphoid organs, and B cells in these organs differ in their functional characteristics and their responsiveness to immunomodulation^12^, we also investigated human splenic B cells. We found an increase in the proportion of B cells that expressed CD25 following stimulation with CpG and CD40L, from 7% at baseline to 28% post-stimulation (*p* < 0.001, Fig. 1c). Similarly, in murine splenic B cells, CD25 expression significantly increased following stimulation with TLR ligands and CD40 ligation (Fig. S1b). Together these data show that at rest, a small proportion of circulating and splenic B cells express the high affinity IL-2R, but that following activation, upregulation of CD25 expression substantially increases this number.

### Effect of IL-2 exposure on B cells

We next asked how the addition of IL-2 would impact B-cell cytokine production following activation in vitro. Stimulation of human splenic B cells with IL-2 alone did not induce secretion of IL-10, IL-6, or TNFα (Fig. 1d–f), but addition of LD IL-2 to CpG- or CD40L-stimulated B cells led to a 6-fold augmentation of IL-10 production (Fig. 1d). In contrast, IL-6 and TNFα production was not affected by IL-2 (Fig. 1e, f) demonstrating that in human B cells, IL-2 skews their cytokine profile towards immunoregulation. We observed that even at very low concentrations of CpG, IL-2 augmented IL-10 production in peripheral blood B cells (Fig. 1g). Similarly, in murine splenic B cells, the addition of IL-2 enhanced IL-10 production in CpG stimulated B cells (Fig. S1c), confirming its capacity to promote the induction of IL-10-producing B cells in vitro.

To assess whether IL-2-mediated augmentation of IL-10-producing B cells would enhance their regulatory capacity, we activated human B cells in vitro in the presence or absence of IL-2, and subsequently co-cultured them with allogeneic CD4 T cells. IL-2-treated B cells significantly suppressed CD4^+^ T cell TNFα production (Figs. 1h, S1d), suggesting that IL-2 promotes the development of IL-10-producing B cells with the functional capacity to regulate CD4^+^ T cells.

### Effect of low dose IL-2 in vivo

**Fig. 2:**
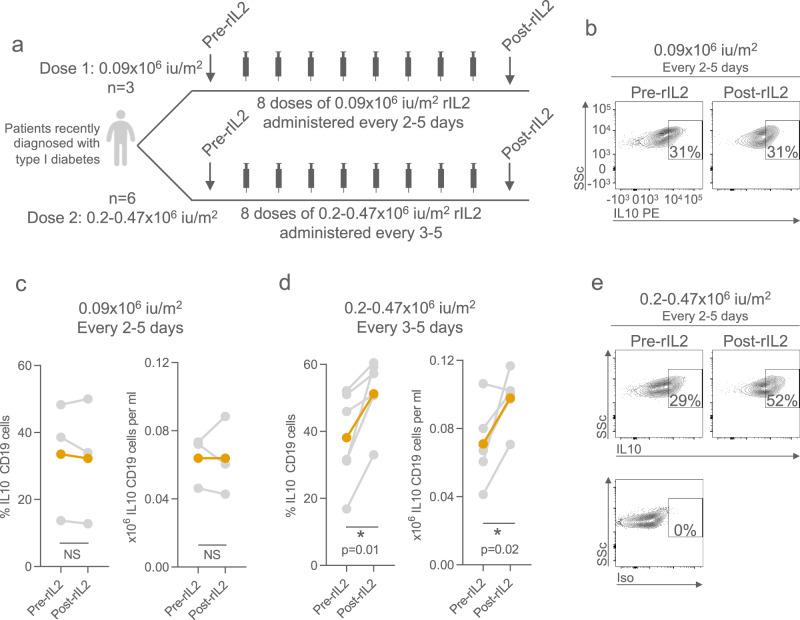
Analysis of B cells and plasma cells from patients given LD IL-2 treatment. **a** Diagrammatic schema summarizing the two treatment arms of the LD aldesleukin study. Patients with recently-diagnosed type 1 diabetes received 0.09 × 10^6^ IU/m^2^ or 0.2–0.47 × 10^6^ IU/m^2^ aldesleukin every two to five days. Proportion (of total B cells) or absolute number of IL-10-positive B cells in patients prior to and following the administration of 0.09 × 10^6^ IU/m^2^ (**c**) or 0.2–0.47 × 10^6^ IU/m^2^ (**d**) aldesleukin. Coloured line indicates mean changes following treatment. Representative flow cytometry plot of IL-10 expressing B cells pre- and post- 0.09 × 10^6^ IU/m^2^ (**b**) or 0.2–0.47 × 10^6^ IU/m^2^ (**e**) aldesleukin. *p*-values generated using a paired two-tailed parametric t-test. *p < 0.05, **p < 0.01, ***p < 0.001. Source data are provided as a Source Data file.

To determine the relevance of these observations to the generation of Bregs in humans in vivo, we analysed peripheral blood mononuclear cells (PBMCs) from patients enrolled in the Adaptive Study of IL2 Dose Frequency on Regulatory T Cells in Type 1 Diabetes (DILfrequency) study (ClinicalTrials.gov NCT02265809)^13,14^. The participants enrolled in this trial were aged between 18 and 70 years and had been diagnosed with type 1 diabetes within 60 months of enrolment. Patients were treated with a variety of doses (0.09–0.47 × 10^6^ IU/m^2^) of the recombinant IL-2 (rIL-2) aldesleukin every two to five days. We chose to analyse samples taken from subjects given the lowest aldesleukin dose of 0.09 × 10^6^ IU/m^2^ as a control, and from subjects given the higher dose ranges of 0.2–0.47 × 10^6^ IU/m^2^ (Figs. 2a, S2a). Overall, there was no change in peripheral B cell counts between samples taken prior to rIL-2 administration and those taken at visit 10 (after 8 doses), 20–50 days after commencing treatment in either group (Fig. S2b). However, in participants who received 0.2–0.47 × 10^6^ IU/m^2^ rIL-2 we observed an increase in the number and proportion of IL-10-producing B cells in contrast to those receiving a 0.09 × 10^6^ IU/m^2^ dose of rIL-2 (*p* < 0.05, Fig. 2b–e).

**Fig. 3:**
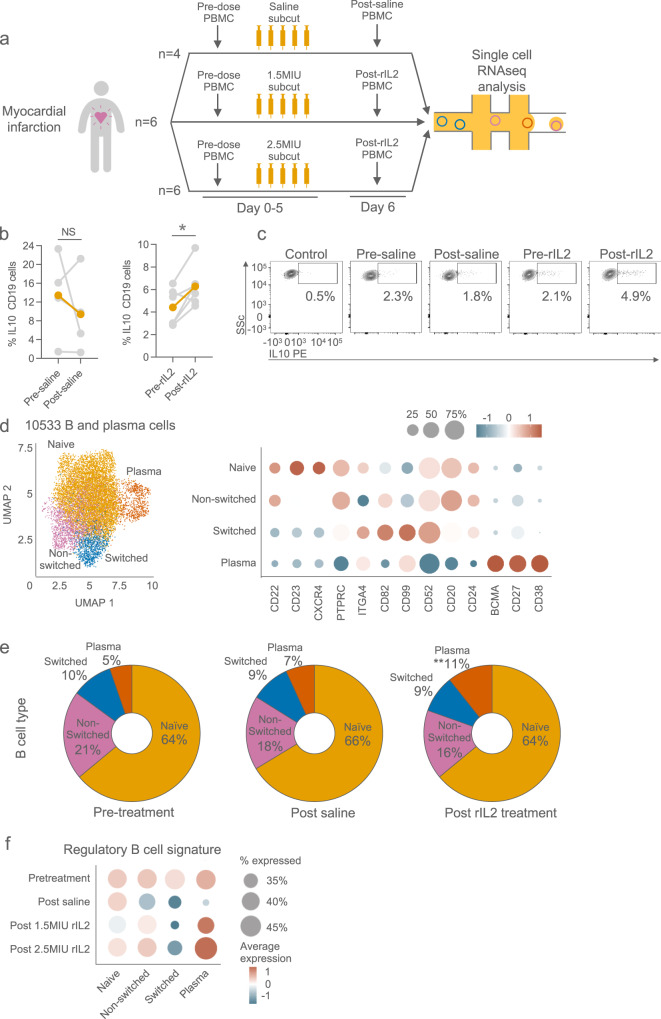
In-depth analysis of B cells and plasma cells from patients given LD IL-2 treatment. **a** Diagrammatic schema summarising the three treatment groups of the trial. Patients received either saline, or 1.5 × 10^6^ IU, or 2.5 × 10^6^ IU IL-2. **b** Proportion of IL-10-positive B cells in patients prior to and following the administration saline (left) or 1.5 × 10^6^ IU IL-2 (right). *n* = 4 and 6 analysed by flow cytometry of untreated and treated groups respectively. *p*-values generated using a paired two-tailed parametric t-test. *p < 0.05. **c** Representative flow cytometry plot of IL-10 expressing B cells pre- and post-LD IL-2. **d** Uniform manifold approximation and projection of four clusters of all B and plasma cells from all samples analysed by scRNAseq: Naïve, non-switched memory and switched memory B cells and plasma cells (left). Dot plot of enriched identifying genes of each cluster (right). **e** Proportion of each cell type before treatment and following saline or LD IL-2 treatment. LD IL-2 treatment groups combined. *p*-values generated using an unpaired two-tailed parametric *t*-test. **p < 0.01 (**f**) Dotplot showing Breg gene set enrichment (gene signatures from recently published meta-analysis of all human Breg RNAseq studies). The intensity of gene signature expression is indicated by the colour of the dot and proportion of cells expressing this signature is indicated by size of the dot. Source data are provided as a Source Data file.

To validate the observed effects of LD IL2 in vivo beyond patients with type I diabetes, we examined PBMCs taken from patients treated with LD aldesleukin as part of the Low-dose Interleukin-2 in Patients with Stable Ischaemic Heart Disease and Acute Coronary Syndromes (LILACS) trial (ClinicalTrials.gov NCT03113773)^15^. In this trial, patients who presented to hospital with a non-ST-elevation myocardial infarction or unstable angina were randomised, within 8 days of presentation to the hospital, to either receive five daily doses of placebo (saline), 1.5 MIU or 2.5 MIU (approximately 0.83 × 10^6^ or 1.39 × 10^6^ IU/m^2^ respectively in a typical 1.8 m^2^ subject) of aldesleukin given subcutaneously (Fig. 3a). Following ex vivo stimulation of PBMCs, we again observed a significant increase in IL-10 producing B cells in patients who were given the 1.5 MIU dose of rIL-2, in contrast to saline-treated controls (Fig. 3b, c).

To better understand the mechanisms by which B cells take on a regulatory phenotype, we performed single-cell RNA sequencing (scRNA-seq) on PBMCs obtained from 16 patients in the LILACS trial before and after LD IL2 treatment. Following standard quality control steps, data on 10,533 B and plasma cells was available and clustered into four groups: naïve, non-switched memory, and switched memory B cells and plasma cells according to canonical marker expression (Figs. 3d and S3a); for example, naïve B cells expressed *CD22* and *CD23*, non-switched memory B cells *PTPRC*, switched memory B cells *ITGA4* and *CD82*, and plasma cells expressed *BCMA*. Cluster annotation was validated by confirming transcriptional similarity to reference datasets (Fig. S3b). Following aldesleukin treatment, there was an increase in plasma cell proportion in contrast to saline-treated subjects (*p* < 0.01, Fig. 3e). There was no change in the immunoglobulin isotype usage in any of the treatment groups (Fig. S3c).

Previous studies have implicated plasma cells as the major source of IL-10 within the B-cell compartment in vivo in some contexts, applying the term Preg^16^. Analysis of cytokine transcripts between saline and aldesleukin treated subjects showed low levels of most cytokines (Fig. S5a) and although serum IL-10 levels were increased post treatment (data not shown), *IL10* transcripts were undetectable in all cell types in our single-cell dataset, including Tregs (in-depth analysis described in a parallel manuscript Zhao et al. under review). This is not unexpected, as droplet-based scRNA-seq methods do not enable the detection of low-level transcripts, and published *10x Genomics* scRNA datasets rarely identify IL-10 transcripts in human cells. Therefore, to address the question of whether in vivo rIL-2 treatment might promote the generation of B cells with regulatory capacity using our scRNA-seq dataset, we assessed for enrichment of a recently curated ‘regulatory B cell’ transcriptional signature^17^. Aldesleukin-treated plasma cells had the highest enrichment of this signature, which was particularly prominent in the 2.5 × 10^6^ IU aldesleukin group, but naïve and non-switched memory B cells in aldesleukin-treated patients also enriched for this regulatory B cell signature compared to saline-treated subjects (Fig. 3f).

### BACH2 and its involvement in Bregs

**Fig. 4:**
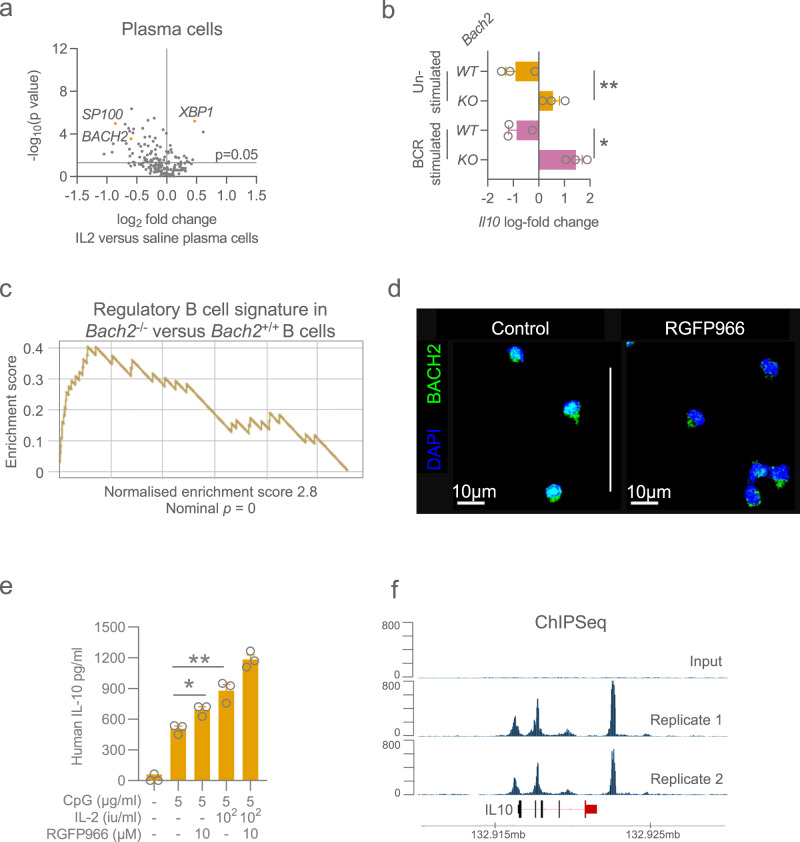
BACH2 inhibits IL-10 production in B cells. **a** Volcano plots of relative transcription factors expression following LD IL-2 treatment versus following saline treatment in plasma cells. *p*-values generated using an unpaired two-tailed parametric t-test. **b** Fold change in IL-10 gene expression comparing murine *Bach2*^−/−^ versus *Bach2*^+/+^ B cells from publicly available data. **c** escarpment plots showing gene set enrichment analysis using Breg gene signature comparing murine *Bach2*^−/−^ versus *Bach2*^+/+^ B cells from publicly available data. **d** Confocal imaging of BACH2 in B cells stimulated with RGFP966, an HDAC3 inhibitor. **e** Quantification of IL-10 in culture supernatants from human blood B cells stimulated with CpG, IL-2 and/or RGFP966, an HDAC3 inhibitor. *p*-values generated using an unpaired two-tailed parametric t-test. *p < 0.05, **p < 0.01. **f** CHIP sequencing data of BACH2 on the *Il10* region of DNA from publicly available data. B cells were unstimulated. Bar graphs show mean and SEM of duplicates. Source data are provided as a Source Data file.

To further delineate the mechanisms by which IL-2 might enhance plasma cell differentiation and regulatory function, we assessed transcription factor (TF) expression in B and plasma cell subsets in saline- and aldesleukin-treated patients (Figs. 4a, S5b). In plasma cells, 17 TFs were differentially expressed with aldesleukin treatment and among those significantly downregulated was *BACH2*, a key TF involved in the differentiation of plasma cells. BACH2 is a transcriptional repressor, and its down-regulation enables the expression of BLIMP-1, required for the terminal differentiation of plasma cells^18,19^. Previous studies have shown that IL-2 treatment alters MAPK–ERK pathway activity, leading to suppression of genes that promote B cell differentiation to plasma cells, including BACH2^20^. Furthermore, since *Bach2-*deficiency in mice is associated with an increase in *Il10* transcripts in CD4 regulatory T cells^21^, we hypothesized that IL-10 production in activated B cells may also be inhibited by BACH2. Consistent with this, we found increased expression of *Il10* transcripts in murine *Bach2*-deficient B cells compared with WT B cells, both in homeostasis and following BCR cross-linking (Fig. 4b). Furthermore, a Breg reference gene signature was enriched in *Bach2*-deficient B cells (Figs. 4c, S6), including increased expression of the co-inhibitory molecules *CTLA4* and *LAG3* (Fig. S7). To confirm these findings in human B cells, we used a highly selective histone deacetylase 3 (HDAC3) inhibitor, RGFP966, previously used to inhibit BACH2 function^22^, given its known dependence on HDAC3^23,24^. Indeed, we observed decreased BACH2 expression and nuclear localisation in peripheral blood B cells in the presence of RGFP966 (Figs. 4d, S8), which significantly augmented IL-10 production in CpG stimulated human B cells (Fig. 4e). Finally, analysis of CHIP-seq data^25^ confirmed Bach2 binding to the Il10 promoter (Fig. 4f). Together, our results suggest that BACH2 directly represses IL-10 expression in B cells, and that IL-2 treatment promotes IL-10 production via BACH2 suppression (Fig. 4g).

Our study indicates that CD25 expression on murine and human B cells increases following activation, generating a high affinity IL-2 receptor that enables responses to low concentrations of IL-2 (10–100 IU/ml). The addition of LD IL-2 to in vitro activated B cells augmented the production of the regulatory cytokine IL-10 without increasing pro-inflammatory cytokines such as IL-6 and TNF. In vivo, we found that the administration of LD aldesleukin to patients increased the number of circulating IL-10-producing B cells, and B and plasma cell enrichment for a regulatory B-cell transcriptional signature. Our scRNA-seq analysis showed that the most significant changes in cell subset proportion post-LD aldesleukin was in plasma cells, which showed increased *Bach2*-downregulation compared to saline treatment. *Bach2* is a transcriptional repressor, and we found it directly binding to the IL-10 gene promoter, and in its absence, B cells have increased *IL10* transcripts and enrichment of a regulatory B cell gene signature, indicating *Bach2* suppression as an important mechanism by which IL-2 may promote an IL-10-producing immunoregulatory phenotype in B cells.

Our data has a number of important implications. Firstly, deficiency of IL-2, CD25, and CD122 have been shown to result in life-threatening autoimmunity in mice and humans^26–29^. This has been assumed to be primarily due to defects in the generation and maintenance of Tregs. However, these historic studies largely utilised global rather than T cell-specific gene knockouts/mutants^26–28^. Therefore, it is possible that reduced generation of Bregs may also contribute to these observed phenotypes. Secondly, LD IL-2 has shown potential therapeutic efficacy in a number of immune-mediated disorders^1–6^. Our study challenges the previous assumption that its immunoregulatory effects are solely mediated by T cells and suggests that Bregs should also be assessed in future trials.

## Methods

All research received ethical approval. Use of splenic tissue: REC reference 15/EE/0152 (East of England - Cambridge South Research Ethics Committee). DILFrequency trial: REC reference 14/EE/1057 (East of England - Cambridge East Research Ethics Committee). LILACS trial: REC reference 17/NW/0012 (North West - Greater Manchester Central Research Ethics Committee). All other experiments involving human tissue are covered under REC reference 12/EE/0446(East of England - Cambridge East Research Ethics Committee). Informed consent was gained from all trial participants.

### Statistics & Reproducibility

Sample size of flow cytometry analysis and scRNAseq of samples from the DILFrequency and LILACS trial were chosen to reflect reasonable minimum numbers required to achieve statistical significance. Samples were excluded only in the case of experiment failure, for example, if frozen cells could not be reanimated. The investigator conducting the experiment was blinded until the analysis was complete. Unless otherwise stated, analysis was done in Microsoft Excel for Mac version 16.61 and Graphpad Prism versions 7 and 9.

### Human peripheral blood B cell CD25 expression

Discarded human leucocyte cones from healthy blood donors were obtained from the National Health Service Blood and Transplant, Cambridge. Leucocytes were diluted in an ice-cold running buffer (produced in-house: 0.5% bovine serum albumin (Sigma-Aldrich), 0.4% 0.5 M pH 8.0 ethylenediaminetetraacetic acid in phosphate buffered solution) and isolated using a Histopaque-1077 (Sigma) density gradient. Cells were washed twice and resuspended in Roswell Park Memorial Institute Solution 1640 (RPMI) supplemented with 10% fetal calf serum, 1% penicillin and streptomycin (all Sigma) in concentrations no greater than 10^6^ cells per 200 μl of supernatant. Cells were stimulated overnight with CpG ODN 2395 (5 ug/ml, InvivoGen) and/or CD40 ligand (1 ug/ml, Peprotech). Cells were washed with PBS and blocked for non-specific antibody binding using 1% normal rat serum (ThermoFisher) and 1% human FcR block (Miltenyi Biotec) for 30 min. Cells were stained with primary surface antibodies for 30 min at room temperature (Table 1). Cells were washed and stained with a viability stain as per the manufacturer’s instructions. Cells were preserved in fixation fluid (produced in-house). Stained leucocytes were processed on a BD LSRFortessa flow cytometer using BD FACSDiva version 6.2 software. Flow cytometry files were analysed on FlowJo version 10 software. Details of all human antibodies used are listed below.

**Table 1:** Human antibodies used in flow cytometry experiments

Antigen	Clone	Chromophore	Manufacturer	Catalog number	Concentration
CD19	HIB19	BV650	BioLegend	302238	5 ul per sample
CD25	M-A251	APC	BD Biosciences	555434	20 ul per sample
Isotype	MOPC-21	APC	BD Biosciences	554681	1 ul per sample
CD25	2a3	APC	BD Biosciences	340939	5 ul per sample
Isotype	X40	APC	BD Biosciences	567155	1 ul per sample
IL-10	JES3-9D7	PE	BioLegend	501404	5 ul per sample
TNFα	Mab11	APC	BioLegend	502912	5 ul per sample
Viability	Live/Dead	Aqua	ThermoFisher	L34957	1:300

### Human spleen B cell CD25 expression and IL-10 production

Human spleen was retrieved from cadaveric organ transplant donors whose organs were turned down for transplantation. Splenic tissue was cut into small pieces and mechanically pushed through a 100-micron metal filter with cRPMI twice. The cell suspension was centrifuged through a histopaque gradient and washed twice. For experiments involving cell surface expression of the IL2 receptor, cells were stimulated, stained, and analysed as described above for peripheral blood leucocytes.

In experiments involving B cell isolation, the splenocytes were subsequently resuspended in running buffer. B cells were magnetically negatively isolated (Miltenyi) as per the manufacturer’s instruction. B cells were placed in a round-bottomed, 96-well plate in 200 ul of supernatant at 5 × 10^5^ cells per well. Cells were stimulated with a combination of CD40 ligand, CpG, or rIL-2 (aldesleukin) for 60 h. In similar experiments analysing effects of IL-2 on lower doses of CpG, blood B cells were isolated from blood leucocyte cones and each well contained 2.5 × 10^5^ cells. Cells were cultured for 72 h. In experiments analysing the effects of HDAC3 inhibition, blood B cells from leucocyte cones were also used and each well contained 5 × 10^5^ B cells and cultured for 60 h. Supernatants were removed and analysed for cytokine concentration by ELISA (R&D Systems) as per the manufacturer’s instructions using BMG CLARIOstar version 5.01 and BMG MARS version 3.01 software.

For co-culture experiments, B cells and CD4 T cells were negatively isolated (Miltenyi) as per the manufacturer’s instructions. B cells were stimulated overnight with CpG, IL-2 or a combination of ligands, and T cells were stimulated overnight on an anti-CD3 antibody (OKT3, Abcam, ab86883)-coated 96-well flat-bottomed plate. Cells were subsequently washed three times to eliminate any residual IL-2 (estimated dilution: 2.7 × 10^4^-fold) and co-cultured overnight at a ratio of 1:5 B cells to CD4 T cells. For the last five hours of co-culture, cells were exposed to PMA (Sigma), ionomycin (Sigma) and brefeldin A (Biolegend) and subsequently stained, fixed, permeabilised and intracellularly stained as per human blood leucocyte samples described below.

### rIL2-treated human blood leucocytes intracellular staining

Details of the methods of the DILFrequency trial have been described elsewhere^13^. In brief, the trial was a mechanistic, non-randomised, repeat dose, open-label, response-adaptive study of 36 consented participants with type I diabetes mellitus using varying doses and frequencies of low-dose rIL-2 intended to augment Treg populations (UK ethics REC reference 14/EE/1057). In our study, we chose a look at a selection of patients from the study with rIL2 doses varying between 0.09 x 10^6^ iu/m^2^ to 0.47 × 10^6^ iu/m^2^ administered every 2 to 5 days. These doses reflect the full range of doses given. Analysed blood samples were taken before rIL-2 treatment (visit 2) and at the beginning of every other visit thereafter (visits 4, 6, 8, and 10).

Retrieved leucocyte samples were defrosted and washed in cRPMI before being rested for 24 h at a concentration of approximately 8 × 10^5^ cells per 200 μl well. Cells were stimulated with CpG ODN 2395 (10 ug/ml) for 48 h. During the last 5 h of this stimulation, cells were additionally exposed to phorbol myristate acetate, ionomycin, and brefeldin. Prior to primary antibody staining, cells were blocked for non-specific binding with normal rat serum and FcR block as described above. Extracellular antibodies were applied as described above. Cells were fixed and stored overnight at 4 °C and permeabilised the following day using an intracellular staining kit as per the manufacturer’s instructions (eBioscience). Intracellular stains were applied for 1 h at room temperature in darkened conditions. Cells were washed and analysed on a flow cytometer. All resting and stimulation of cells took place in a 96-well round-bottomed plate in an incubator at 36 °C and 5% CO_2_. All samples were processed in duplicate along with an unstimulated isotype control. The gating strategy is shown in Fig. S9.

Blood samples from the LILACS study were analysed in a similar fashion to the DILFrequency trial. Duration of CpG stimulation and PMA, ionomycin and brefeldin exposure were reduced to 36 and 4 h respectively. Sex and gender were considered in the study design. Sex was assigned as per medical records. Samples groups that were analyzed by flow cytometry were derived from 4 males in the saline group and 3 males and 3 females in the IL-2 treated group.

### Murine spleen B cell CD25 expression

Mouse spleens were collected from 6 to 12 week-old C57BL/6 wildtype mice bred within the University of Cambridge mouse facilities in specific pathogen-free conditions at a Home Office-approved facility in the UK in line with guidance set out by the National Centre for the Replacement, Refinement and Reduction of Animals in Research with regard to dark/light cycle, ambient temperature and humidity. All procedures were carried out in accordance with the United Kingdom Animals (Scientific Procedures) Act of 1986. Spleens were mechanically pushed through a 70-micron filter with cRPMI. Cells were washed and exposed to red cell lysis solution (produced in-house) and washed again. Methods for en masse stimulation experiments and isolated B cell experiments were as per human splenic experiments except murine CD40L (1 ug/ml, Peprotech) and LPS (10 ug/ml, Sigma), (Table 2).

**Table 2:** Murine antibodies used in flow cytometry experiments

	Clone	Chromophore	Manufacturer	Catalog number	Concentration
B220	RA3-6B2	APC/Cy7	Biolegend	103224	1:100
CD25	PC61	PerCP/Cy5.5	Biolegend	102030	1:100
Isotype for above	G0114F7	PerCP/Cy5.5	Biolegend	401910	1:100

### Murine spleen B cell IL-10 production

A cell suspension was generated from whole splenic tissue as described above. B cells were negatively isolated (Miltenyi) as per the manufacturer’s instructions. B cells were placed in a round-bottomed, 96 well plate in 200 ul of supernatant at 5 × 10^5^ cells per well and stimulated with combinations of CpG, CD40L, and IL-2. Supernatant concentrations of IL-10 were analysed by ELISA (R&D Systems).

### RNA-seq data analysis

Genome-wide transcriptional data of IL-10 producing Bregs and *Bach2*^*−/−*^ B cells was obtained from a publicly available dataset (GEO accession number GSE35002 and GSE103982) and analysed through the GEO2R portal^25,30^. A heatmap was generated using heatmaps function from the ggplot2 package.

### Single-cell RNAseq sample processing

Details of the methods of the LILACS trial have been described elsewhere^15^. In brief, the trial was a randomised, single centre trial involving consented patients with acute coronary syndrome given varying doses of low-dose rIL-2 intended to augment Treg populations (UK ethics REC reference 17/NW/0012). Sex and gender were considered in the study design. Samples were derived from 4 males in the saline group, 3 males and 3 females in the 1.5 MIU IL2 group and 4 males and 2 females in the 2.5 MIU IL2 group.PBMCs were removed from −80 storage and placed onto wet ice. The cells were defrosted on ice-cold PBS and suspended in a final volume of 50 ml keeping the sample cool throughout this stage. The cells were centrifuged at 400 × g for 5 min. The supernatant was removed and cells resuspended in a small volume of PBS with the addition of CaCl_2_. Live cells were enriched using Easysep dead cell removal kit as per the manufacturer’s instructions. Cells were centrifuged as before, counted and 10^6^ cells were resuspended in 50 ul of PBS. Each sample was blocked with 10 ul FC block (Miltenyi), stained with 0.5 ul of Total Seq antibody using pre-mixed as a master mix for 30 min at 4 °C. Cells were centrifuged and washed twice as above, counted and diluted to an appropriate concentration for loading on the 10×.

For the 10×, the 5’ kit was used. Samples were loaded on to the chip as per manufactures recommendations with an aim to recover 10,000 cells per lane. Remainder of the 10× library was carried out as per the manufacturer’s instructions and the resulting libraries (GEX, TCR, BCR and CITE) sequenced on a Novaseq 2 × 150 at Genewiz. BCL files were demitiplexed using Casava (Illumina) and count tables produced using Cell ranger (10x genomics).

### Single-cell RNAseq data and preprocessing

The single-cell data (10X cellranger output) was corrected for ambient RNA expression using SoupX (v1.5.0)^31^. SoupX was run with clustering information derived from a generic processing workflow in Seurat^32^. After SoupX, doublet detection was performed using scrublet (v0.2.3)^33^ with adaptations outlined in Popescu et al.^34^. Briefly, after scrublet was performed, the data was iteratively sub-clustered using standard Seurat-inspired scanpy (v.1.7.1) workflow and a median scrublet score for each sub-cluster was computed^32^. Median absolute deviation (MAD) scores were computed from the cluster scrublet scores and a one tailed t-test was performed with Benjamini-Hochberg (BH) correction^35^ applied and cells with significantly outlying cluster scrublet scores (BH *p*-value < 0.1) were flagged as potential doublets. The data was then processed using scanpy with standard quality control steps; cells were filtered if number of genes > 6000 or < 200. The percentage mitochondrial content cut-off was set at < 10%. Genes were retained if they are expressed by at least 3 cells. Genes counts for each cell were normalised to contain a total count equal to the median of total counts in cells before normalisation. This led to a working dataset of 208,750 cells. We filtered out 18,038 cells as potential doublets after manual inspection of the data. Highly variable genes were selected based on the following parameters: minimum and maximum mean expression are ≥ 0.0125 and ≤ 3 respectively; minimum dispersion of genes = 0.5. TCR and BCR V(D)J genes and light chain constant genes were excluded as highly variable genes. The number of principal components used for neighbourhood graph construction and dimensional reduction was set at 50. Batch correction was performed using harmony with subjects as the batch term with all other parameters as per default settings. Clustering was performed using Leiden algorithm^36^. In all cases where Uniform Manifold Approximation and Projection (UMAP; v3.10.0)^37^ was used for dimensional reduction and visualisation, the minimum distance was set at 0.3 and all other parameters as per default settings in scanpy.

### Automatic cell-type annotation

B cell subgroups (eg naive, switched etc.) were manually determined using differentially expressed B cell markers^38,39^. To confirm the identity of each cell, Reference-Based Single-Cell RNA-Seq Annotation (SingleR v. 3.13) was employed (Fig. S3b)^40^. B cell subtype calls were made using the Singler function, and Seurat’s WhichCells and DimPlot functions were used for graphing. Although a number of different datasets could be applied to this technique (eg. Human Primary Cell Atlas, Bluepoint Encode Data or Novershtem Hematopoietic Data), all returned similar results (data not shown). UMAP generated using the MonacoImmuneData shown^41^.

### Breg geneset enrichment

A list of Breg-enriched genes from a meta-analysis of previous studies was generated by Dubois et al.^17^. The Seurat AddModuleScore function calculated the average expression levels of a set of genes on a single cell level. Employing this data, a dot plot was generated using the Seurat DotPlot function.

### Transcription factors

An unbiased list of major transcription factors compiled by Transcriptional Regulatory Relationships Unraveled by Sentence-based Text mining (TRRUST; www.grnpedia.org/trrust/) was analysed between LD IL-2 treated and saline-treated B cells in the four B cell subtypes^42^. Comparisons were made using the Seurat FindMarkers function which identify statistically significant differentially expressed genes. Volcano plots were generated using Graphpad Prism version 7 and 9.

### Gene set enrichment analysis

Preranked gene-set analysis of *Bach2*^−/−^ versus *Bach2*^+/+^ using Dubois et al. regulatory B cell gene signature was performed^17,25^. Preranking was performed on the Gene Expression Omnibus 2 R (GEO2R) portal. Genes were preranked according to signed −log_10_ *p* values for all preranked gene-set analysis procedures. GSEA was done using the Subramanian et al. software (version 4.1.0).

### Confocal microscopy

Human blood B cells were negatively isolated and prepared for stimulation as described above and stimulated overnight with control medium containing equivalent concentration of DMSO or RGFP966 (Selleck, 10 uM). B cells were fixed (4% paraformaldehyde), deposited onto poly-lysine slides, permeabilised (Tris 0.1 M, 1% BSA, 1% normal donkey serum, 0.3% triton x-100), stained with 1:50 mouse anti-human HDAC3 (Biorbyt, orb97554) and rabbit anti-human BACH2 (Invitroge, PA5-84376), followed by 1:250 donkey anti-mouse Alexa 555 (Invitrogen, A-31570) and donkey anti-rabbit Alexa 488 (Invitrogen, A-21206) and finally mounted using DAPI fluomount GTM(ThermoFisher). Imaging was carried out using a Zeis-880 confocal microscope in AiryscanTM super-resolution mode at identical settings. Image analysis was carried out on Zeiss Zen and image J version 1.53 software. To quantify nuclear BACH2 regions of interest were created using the DAPI and then overlayed onto BACH2 to calculate the mean nuclear BACH2 intensity in each condition.

### Chip-seq

The ChIP-seq data was downloaded from Gene Expression Omnibus access number GSE87503 and converted from bigWig to bed format with bigWigToWig (v3.77) and wig2bed (BEDOPS v2.4.39) for chromosome 1^25^. Peaks were called with macs2 (v2.2.7.1) with *q* = 0.05 in “nomodel” mode. The data was visualised in GViz bioconductor R package (v1.36.1).

### Reporting summary

Further information on research design is available in the Nature Portfolio Reporting Summary linked to this article.

## Supplementary information

Supplementary Information

Reporting Summary

## Data Availability

IL-10 producing B cell, *Bach2*^−/−^ and Chipseq data are publicly available from the Gene Expression Omnibus: GSE35002, GSE103982, GSE87503 (https://www.ncbi.nlm.nih.gov/geo/query/acc.cgi), scRNA data available on request from Tian X Zhao (txz20@cam.ac.uk). Source data are provided with this paper.

## Source data

Source Data
