# Supplementary material for: Low-dose IL-2 enhances the generation of IL-10-producing immunoregulatory B cells
Source: Nat Commun. 2023 Apr 12;14:2071. doi: 10.1038/s41467-023-37424-w (PMC10097719; doi:10.1038/s41467-023-37424-w)
Supplement: Supplementary file 1 — Supplementary Information [file 41467_2023_37424_MOESM1_ESM.pdf]

Supplementary figure 1

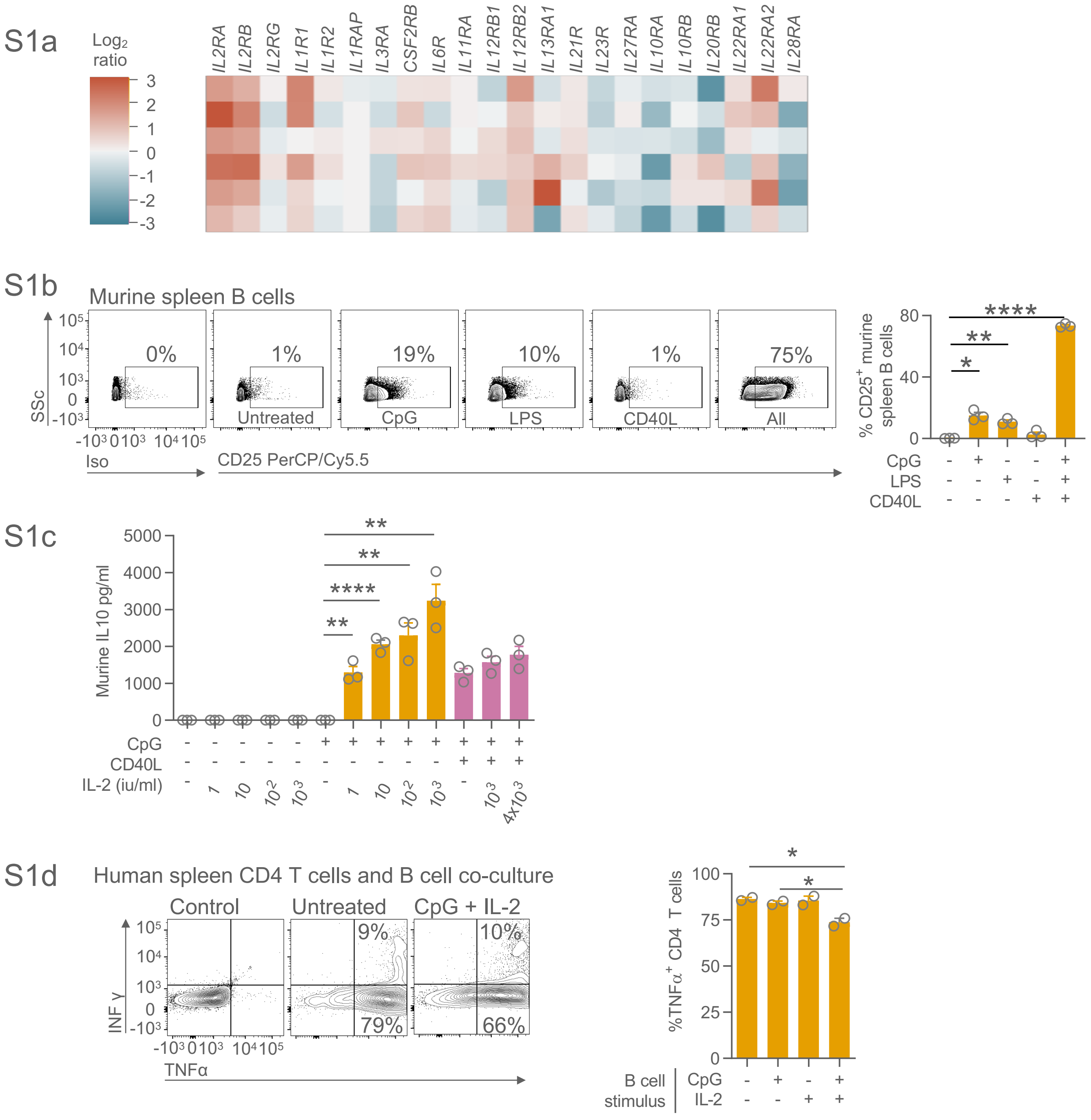

Supplementary figure 1: Activated B cells express CD25 and the addition of IL-2 increases IL-10 production by *in vitro*. **(a)** Extended heatmap showing cytokine receptor transcript expression in IL-10-positive human B cells relative to IL-10-negative B cells from publicly available data. **(b):** Representative FACS plots and quantification of surface expression of CD25 (IL-2Rα) on murine splenic B cells. Gated on live single B220^{+} events. Graphs show means and error bars show standard error of mean (SEM) of triplicates. Representative of three experimental replicates. *p* values generated using an unpaired two-tailed parametric t-test. **(c)**: Quantification of IL-10 in culture supernatants from negatively isolated murine splenic B cells stimulated with IL-2, CpG and/or CD40L. Graph shows means and SEMs of triplicates and are representative of three experimental repeats. p values generated using an unpaired two-tailed parametric t-test. **(d):** Representative FACS plots (left) and quantification (right) of the proportion of TNFα positive human CD4 T cells, as evidenced by intracellular cytokine staining, following co-culture with CpG-stimulated B cells generated in the presence or absence of IL-2. Graphs show mean and SEM of duplicates and are representative of three experimental repeats. p values generated using an unpaired two-tailed parametric t-test. * p<0.05, ** p<0.01, *** p<0.001. Source data are provided as a Source Data file.

Supplementary figure 2

S2a

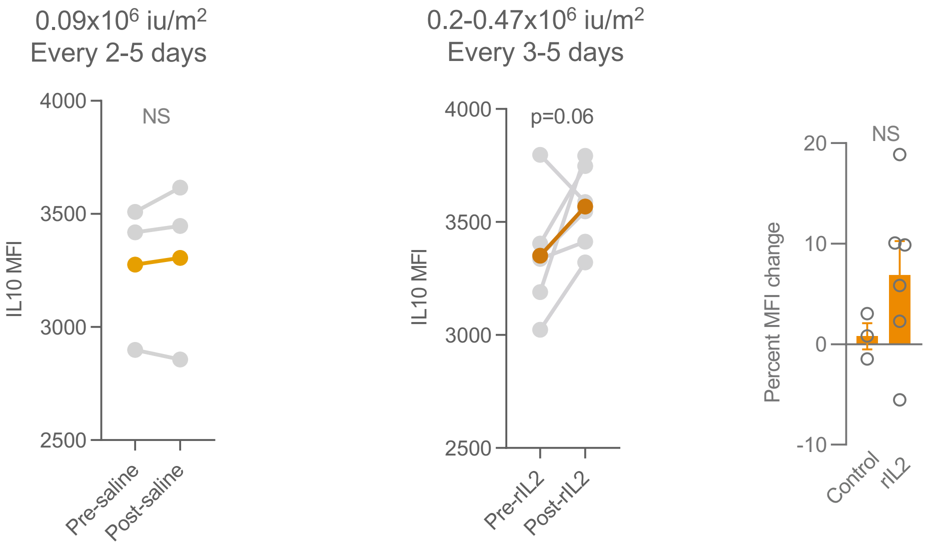

S2b

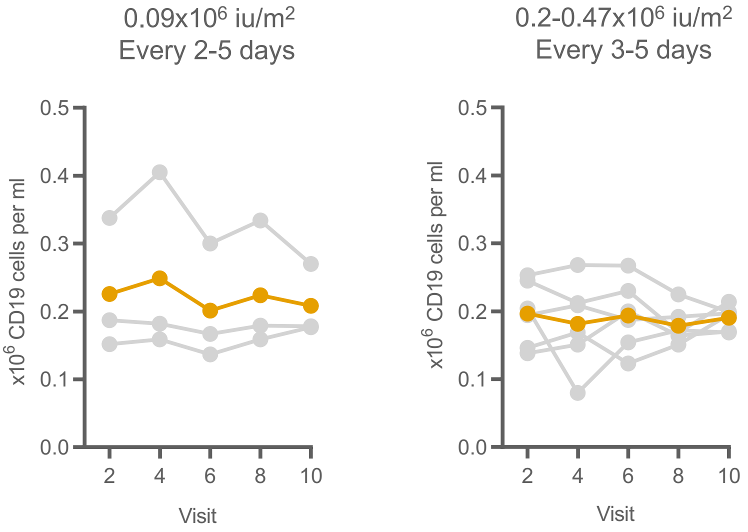

Supplementary figure 2: Analysis of IL-10 producing B cells following IL-2 treatment in humans. **(a)**: Left: B cell IL-10 MFI in patients prior to and following the administration of 0.09x1^{6} IU/m^{2}. Centre: Following the administration of 0.2-0.47x10^{6} IU/m^{2} aldesleukin. Coloured line indicates mean changes following treatment. Right: Percent MFI change following low dose IL-2 treatment. Bar graph shows mean and SEM of duplicates. *p* values generated using a paired two-tailed parametric t-test. NS not significant. **(b)**: Left: Total peripheral blood B cell numbers during rIL-2 treatment. B cell count taken during visits 2, 4, 6, 8 and 10 of DILfrequency trial of patient given 0.09x10^{6}IU/m^{2}. Right: B cell count when given 0.2-0.47x10^{6} IU/m^{2} aldesleukin dose. Coloured line indicates mean values. Source data are provided as a Source Data file.

Supplementary figure 3

S3a

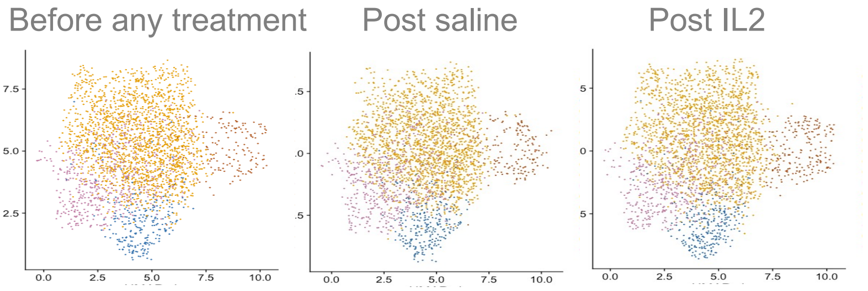

S3b

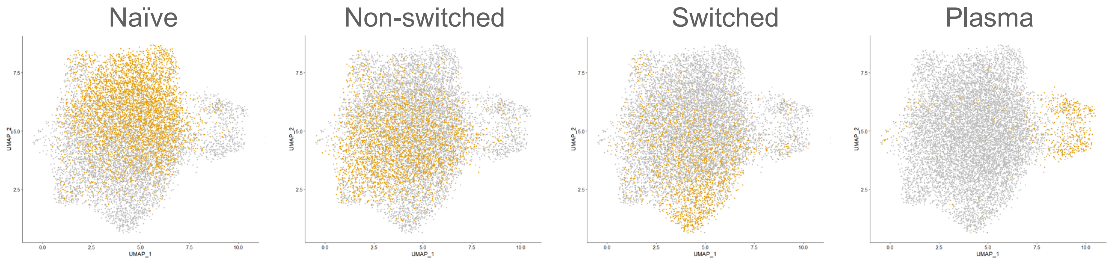

S3c

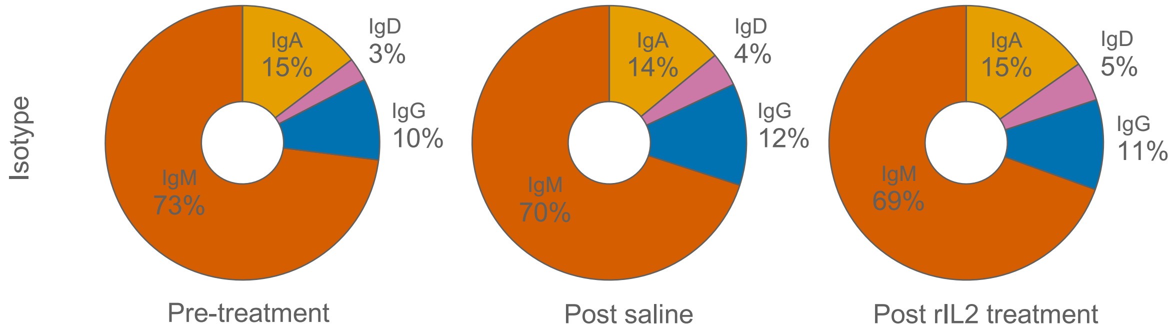

Supplementary figure 3: Analysis of scRNAseq data of B cells from patients receiving low-dose IL-2 treatment. **(a)**: UMAP of cells UMAPS of cells taken pre-treatment, post saline and post IL2. Number of cells adjusted to be equal on each UMAP. **(b):** Cluster identification using reference-based single cell RNA-seq annotation. **(c)**: Proportion of B cell isotype before and after treatment with saline and LD rIL-2**.**

Supplementary figure 4

S4

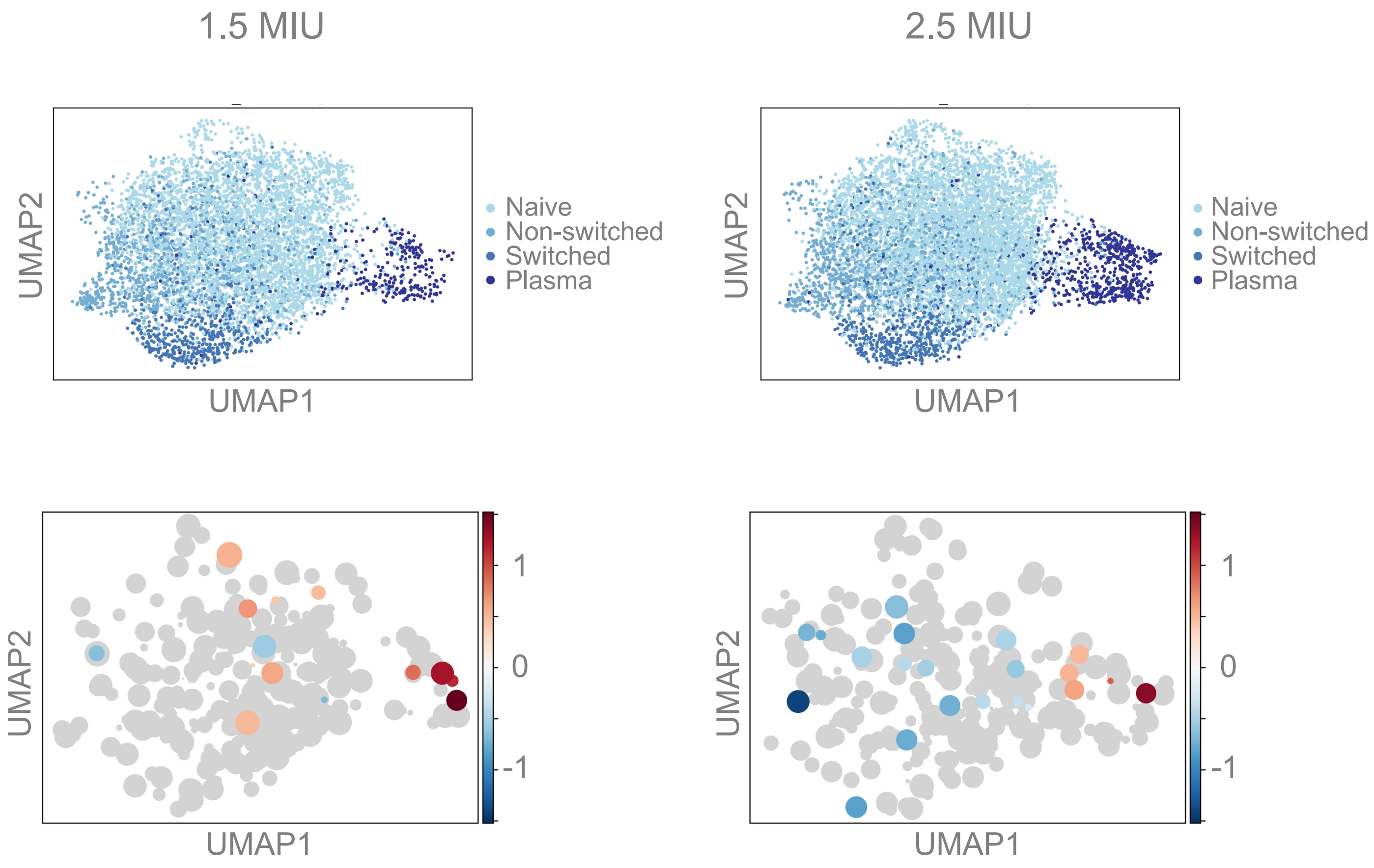

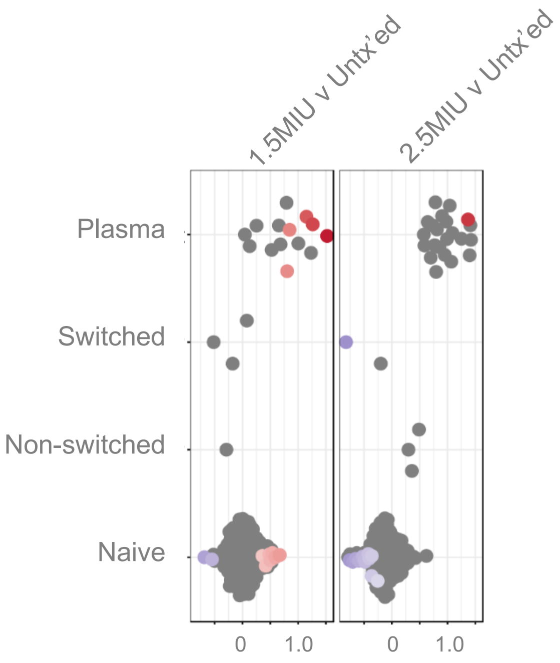

Supplementary figure 4: Differential abundance testing of single-cell neighborhoods between untreated and post-IL-2 treatment groups with a negative binomial generalized linear mixed-effects model (n=20 samples from 10 patients, 4 treated with placebo and 6 with IL-2). The coloured dots on the UMAPs are significantly ($p<0.05$) differentially abundant and the gradient indicates the beta coefficients for each neighbourhood from blue to white to red, where white indicates a value of 0 (no change). Size or dots indicate the size of neighbourhoods. The bottom plot shows the beta coefficients for each neighbourhood assigned to corresponding cell types where positive and negative coefficient values are interpreted as enriched or depleted after IL-2 treatment, respectively. Differentially abundant neighbourhoods are coloured according to the beta coefficient value from blue to white to red, where white indicates a value of 0 (no change). Nonsignificant neighbourhoods are coloured grey. Significance calculated by linear mixed effect model accounting for age and gender with B-H corrected two-tailed p-values.

Supplementary figure 5

S5a

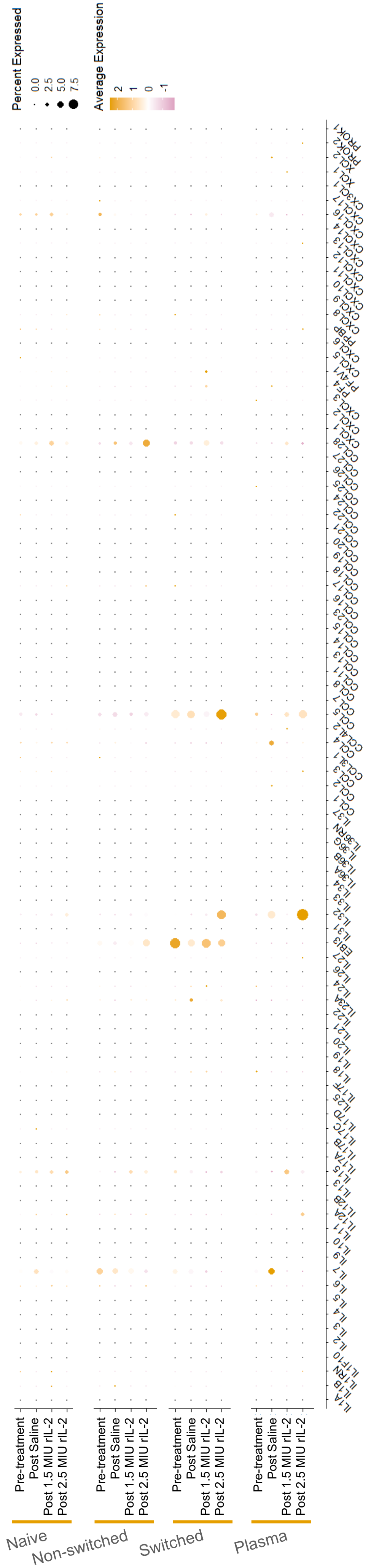

S5b

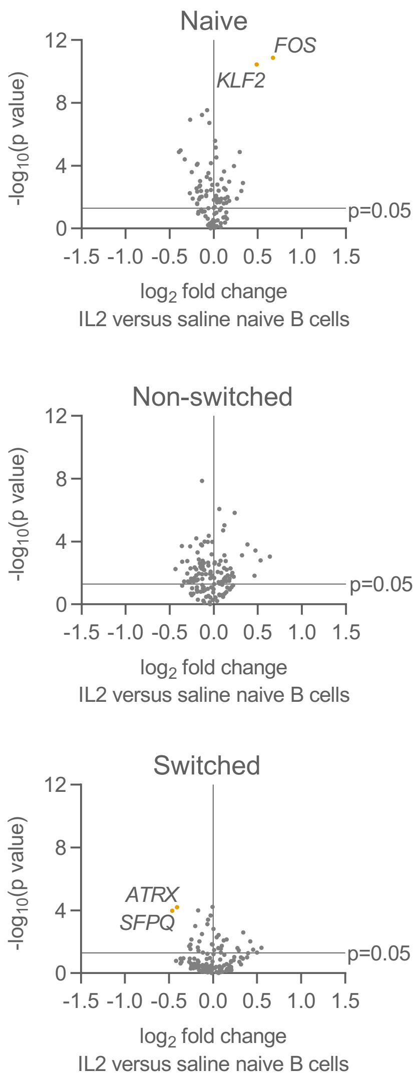

Supplementary figure 5: Analysis of cytokine and transcription factors of scRNAseq data. **(a):** Dot plot of cytokines and interleukins before and after treatment with saline and different doses of LD rIL-2. **(b):** Volcano plots of relative transcription factors following LD IL-2 treatment versus following saline treatment in naïve, non-switched memory and switched memory B cells. Significance calculated by two-sided Wilcoxon rank sum test with with B-H correction.

Supplementary figure 6

S6a

Up-regulated genes
(Unstimulated)

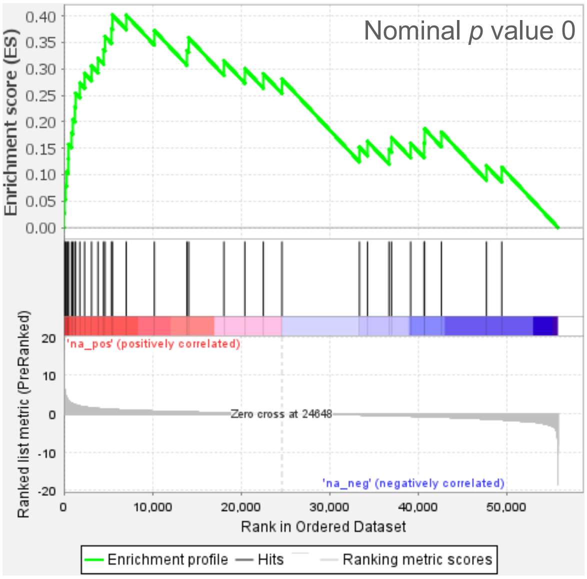

Down-regulated genes
(Unstimulated)

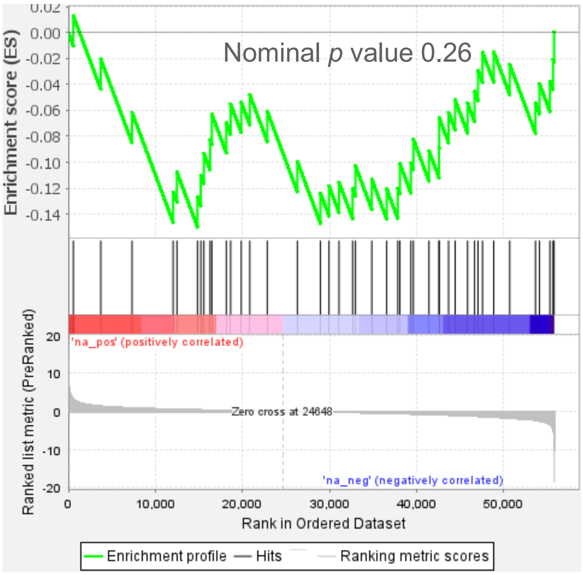

S6b

Up-regulated genes
(BCR-stimulated)

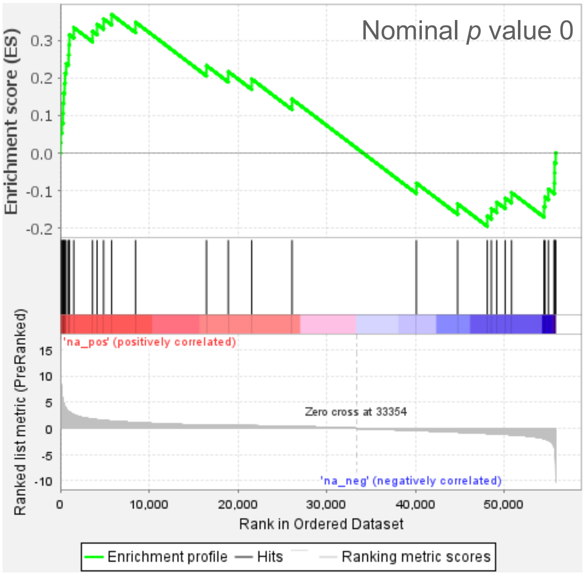

Down-regulated genes
(BCR-stimulated)

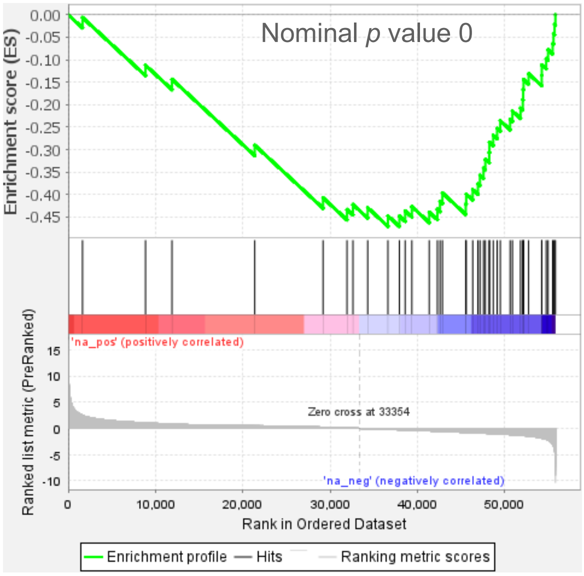

Supplementary figure 6: Gene set enrichment analysis of using up- and downregulated Breg gene set comparing *Bach2*$^{-/-}$ versus *Bach2*$^{+/+}$ B cells without (**a**) and with (**b**) BCR stimulation. Breg gene set derived from recently compiled metanalysis of multiple previous RNAseq studies. Bar graph shows mean and SEM of duplicates. Significance calculated by permutation-based p-value from within GSEA. ** p<0.01.

Supplementary figure 7

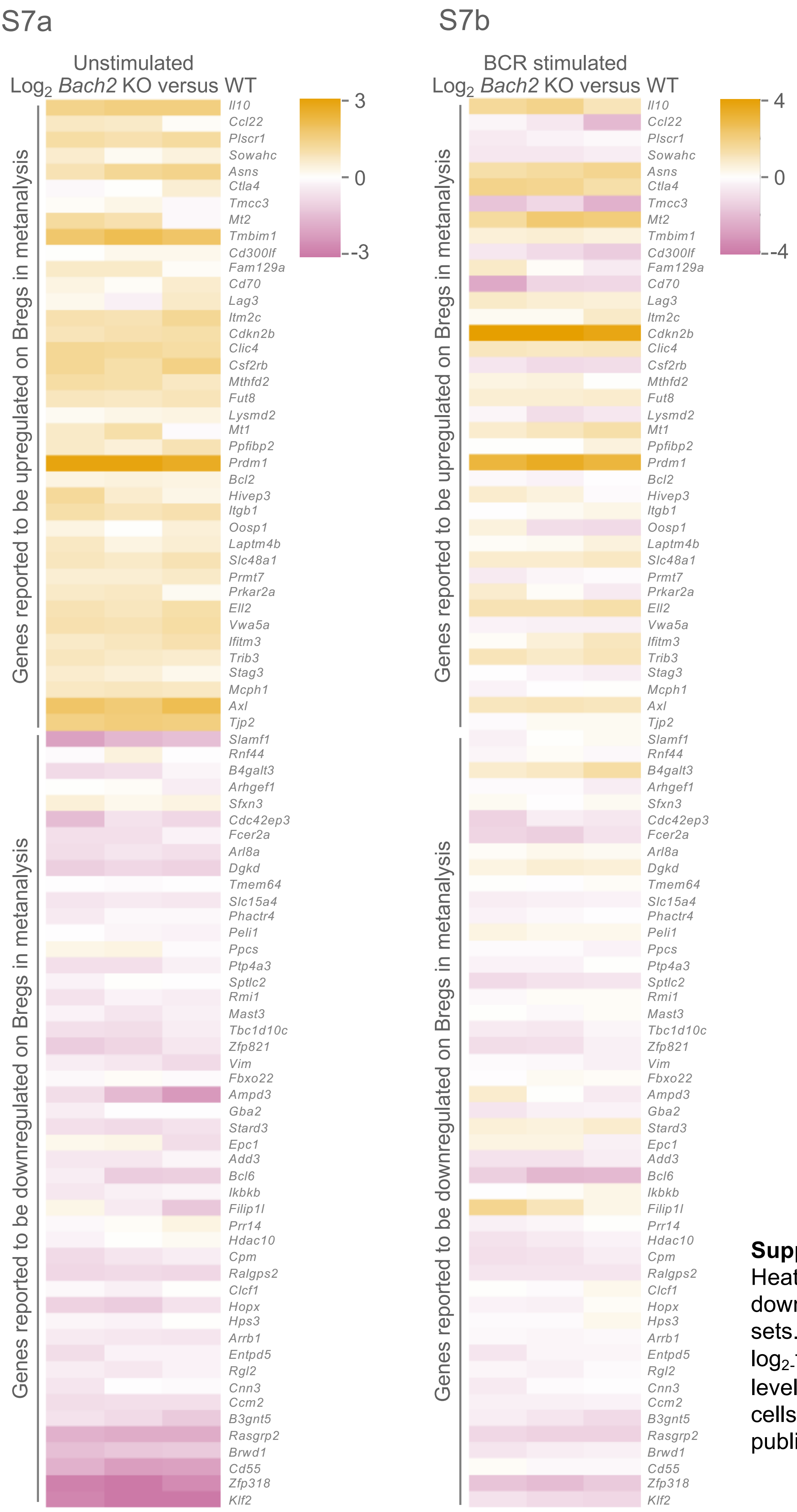

Supplementary figure 7: Heatmap of up- (**a**) and downregulated (**b**) Breg gene sets. Intensity of colour indicates $\log_2$-fold increase in transcript level of Bach$^{-/-}$ over Bach$^{+/+}$ B cells. Figure generated from publicly available data.

Supplementary figure 8

S8a

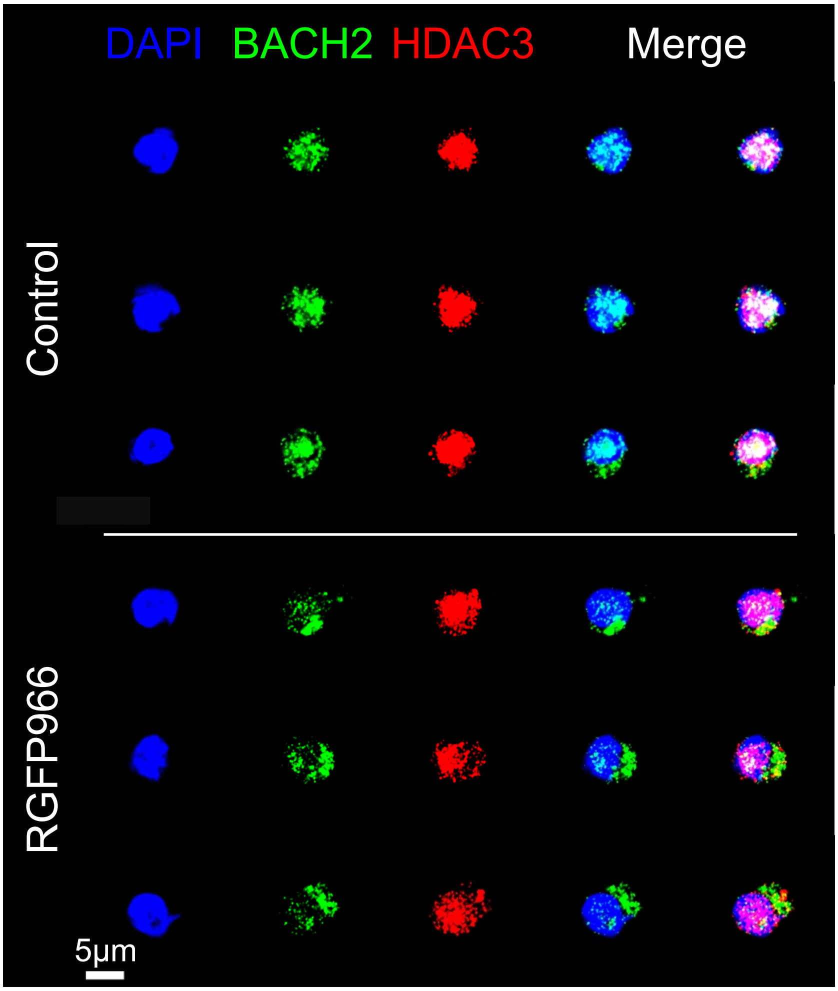

S8b

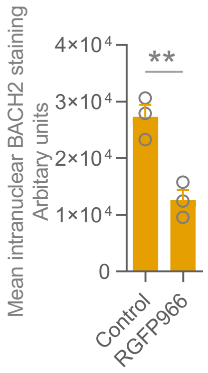

Supplementary figure 8 (a): Zoomed hi-resolution images of individual B cells with reduction intranuclear staining of BACH2 following HDAC3 inhibition compared to control cells. HDAC3 is localised to the nucleus in both control and inhibited B cells. **(b):** Quantification of intranuclear BACH2 staining. p values generated using an unpaired parametric t-test. * p<0.05, ** p<0.01, *** p<0.001. Source data are provided as a Source Data file.

Supplementary figure 9

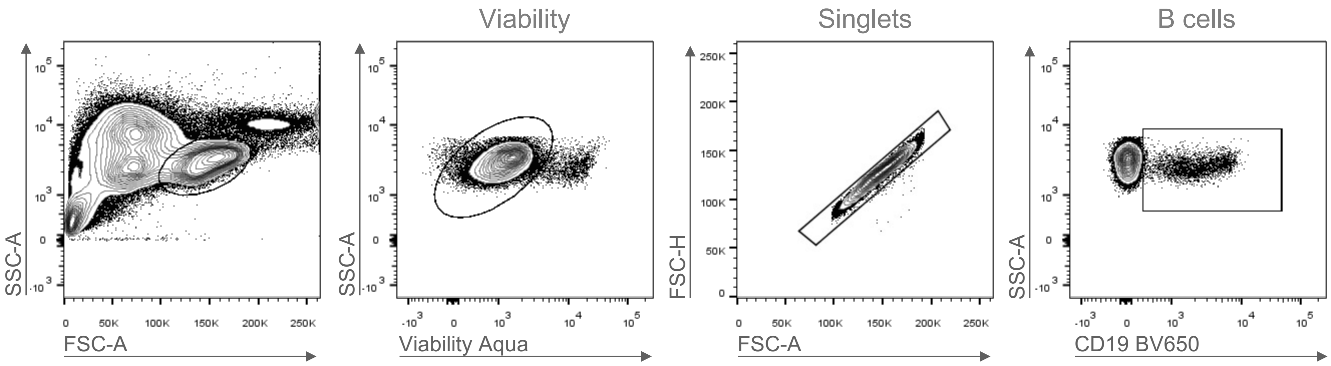

Supplementary figure 9: Gating strategy for human B cells.

Supplementary figure 10

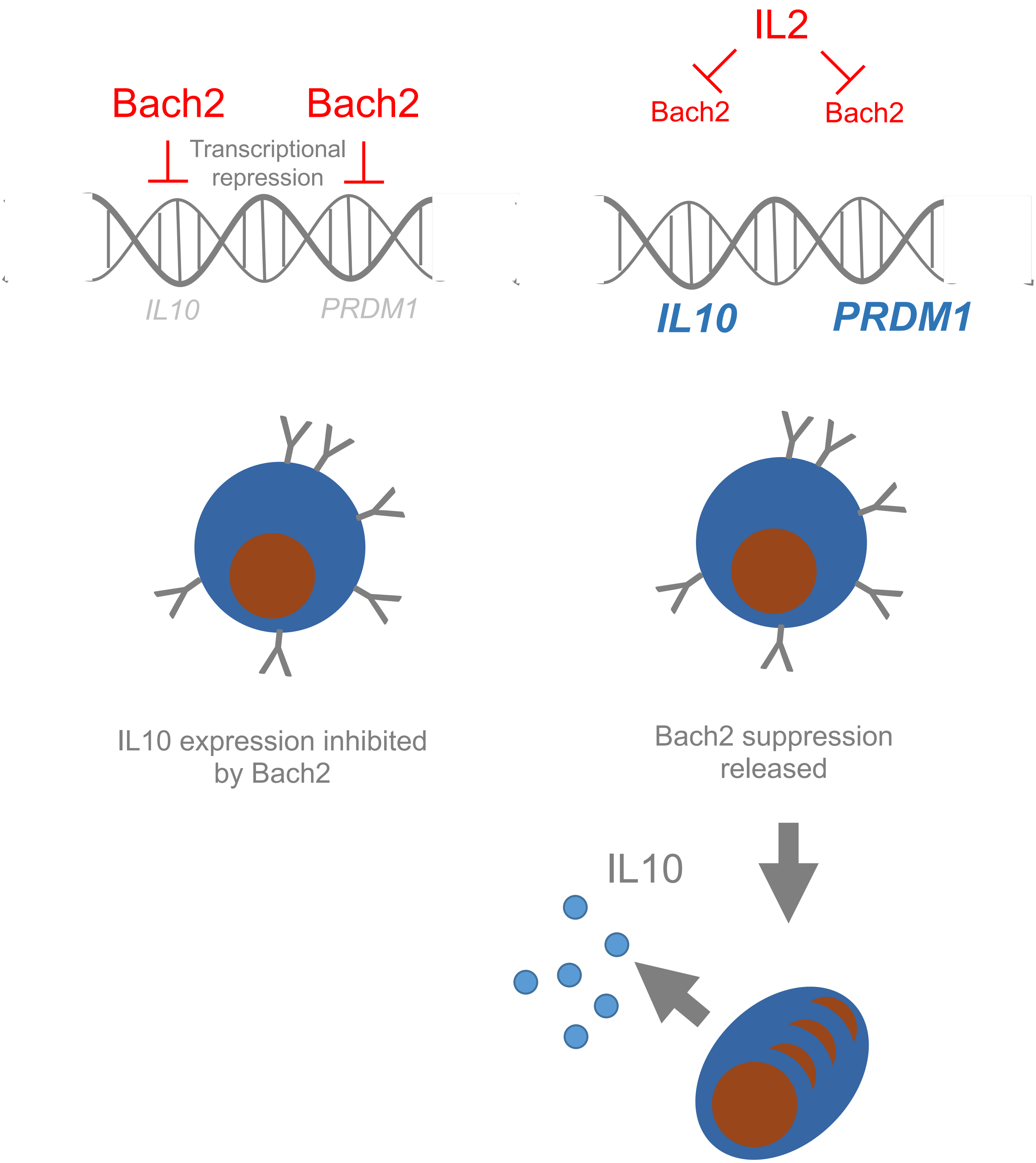

Supplementary figure 10: Graphical summary of the effects of Bach2 on inhibiting IL10 transcription during physiological conditions (left) and the effects of low dose IL2 on inhibiting Bach2 and allowing IL10 transcription (right).
