# Supplementary material for: Low-dose IL-2 enhances the generation of IL-10-producing immunoregulatory B cells
Source: Nat Commun. 2023 Apr 12;14:2071. doi: 10.1038/s41467-023-37424-w (PMC10097719; doi:10.1038/s41467-023-37424-w)
Supplement: Supplementary file 2 — Reporting Summary [file 41467_2023_37424_MOESM2_ESM.pdf]

nature portfolio

Corresponding author(s): Menna Clatworthy

Last updated by author(s): 2022 05 25

Reporting Summary

Nature Portfolio wishes to improve the reproducibility of the work that we publish. This form provides structure for consistency and transparency in reporting. For further information on Nature Portfolio policies, see our Editorial Policies and the Editorial Policy Checklist.

Statistics

For all statistical analyses, confirm that the following items are present in the figure legend, table legend, main text, or Methods section.

n/a	Confirmed	
☐	☒	The exact sample size (*n*) for each experimental group/condition, given as a discrete number and unit of measurement
☒	☐	A statement on whether measurements were taken from distinct samples or whether the same sample was measured repeatedly
☐	☒	The statistical test(s) used AND whether they are one- or two-sided<br>*Only common tests should be described solely by name; describe more complex techniques in the Methods section.*
☒	☐	A description of all covariates tested
☒	☐	A description of any assumptions or corrections, such as tests of normality and adjustment for multiple comparisons
☐	☒	A full description of the statistical parameters including central tendency (e.g. means) or other basic estimates (e.g. regression coefficient) AND variation (e.g. standard deviation) or associated estimates of uncertainty (e.g. confidence intervals)
☐	☒	For null hypothesis testing, the test statistic (e.g. *F*, *t*, *r*) with confidence intervals, effect sizes, degrees of freedom and *P* value noted<br>*Give P values as exact values whenever suitable.*
☒	☐	For Bayesian analysis, information on the choice of priors and Markov chain Monte Carlo settings
☒	☐	For hierarchical and complex designs, identification of the appropriate level for tests and full reporting of outcomes
☒	☐	Estimates of effect sizes (e.g. Cohen's *d*, Pearson's *r*), indicating how they were calculated

Our web collection on statistics for biologists contains articles on many of the points above.

Software and code

Policy information about availability of computer code

Data collection	BD FACSDiva version 6.2 for flow cytometry<br>BMG CLARIOstar version 5.01 for ELISA<br>Novaseq 2x150 at Genewiz
Data analysis	Microsoft Excel for Mac version 16.61<br>Graphpad Prism version 7 and 9<br>FlowJo version 10<br>BMG MARS version 3.01<br>ggplot2 version 3.4<br>Illumina Casava version 1.6<br>10X Genomics Cell Ranger<br>SoupX version 1.5.0<br>Scrublet version 0.2.3<br>Seurat-inspired scanpy version 1.7.1<br>Uniform Manifold Approximation and Projection version 3.10.0<br>SingleR version 3.13<br>Seurat WhichCells version 2.3.1<br>Seurat DimPlot version 2.3.1<br>Seurat AddModuleScore 2.3.1

Gene Expression Omnibus 2R (GEO2R)
Gene Set Enrichment Analysis version 4.1.0
bigWigToWig version 3.77
wig2bed BEDOPS version 2.4.39
macs2 version 2.2.7.1
GViz bioconductor R package 1.36.1
ImageJ version 1.53

For manuscripts utilizing custom algorithms or software that are central to the research but not yet described in published literature, software must be made available to editors and reviewers. We strongly encourage code deposition in a community repository (e.g. GitHub). See the Nature Portfolio guidelines for submitting code & software for further information.

Data

Policy information about availability of data

All manuscripts must include a data availability statement. This statement should provide the following information, where applicable:

- Accession codes, unique identifiers, or web links for publicly available datasets
- A description of any restrictions on data availability
- For clinical datasets or third party data, please ensure that the statement adheres to our policy

IL-10 producing B cell data: GSE35002 (no access restrictions)
BACH2 -/- data: GSE103982 (no access restrictions)
Chipseq data: GSE87503 (no access restrictions)

scRNAseq data generated for the LILACS study is available upon reasonable request. Please contact Tian X Zhao for access.

Human research participants

Policy information about studies involving human research participants and Sex and Gender in Research.

Reporting on sex and gender

DILFrequency:
Sex and gender were considered in the study design. Please see Table 1 of Seelig et al, JCI Insight 2018. Sex was assigned as per medical records.

In this study, DILFrequence samples groups which were analyzed by flow cytometry for Bregs were derived from 3 males in the control group and 4 males and 2 females in the treatment group.

LILACS:
Sex and gender were considered in the study design. Please see Table 1 of Zhao et al, NEJM Evidence 2021. Sex was assigned as per medical records.

In this study, LILACS samples groups which were analyzed by flow cytometry for Bregs were derived from 4 males in the saline group and 3 males and 3 females in the IL-2 treated group. Samples groups which were analyzed by single cell RNA seq were derived from 4 males in the saline group, 3 males and 3 females in the 1.5 MIU IL2 group and 4 males and 2 females in the 2.5 MIU IL2 group.

Population characteristics

DILFrequency:
For population characteristics, please see Table 1 of Seelig et al, JCI Insight 2018.

In this study, DILFrequency samples groups which were analyzed by flow cytometry for Bregs were derived from subjects 28 to 53 years of age in the control group and 34 to 58 in the treatment group.

LILACS:
For population characteristics, please see Table 1 of Zhao et al, NEJM Evidence 2021.

In this study, LILACS samples groups which were analyzed by flow cytometry for Bregs were derived from subjects aged 53-79 years in the saline group and aged 57 to 70 years in the IL-2 treated group. Samples groups which were analyzed by single cell RNA seq were derived from subjects aged 53-79 years, aged 57 to 70 years in the 1.5 MIU IL2 group and aged 43-73 years in the 2.5 MIU IL2 group.

Recruitment

DILFrequency:
Participants were eligible if they were aged 18 to 70 years and had a duration of type I diabetes of ≤5 years from diagnosis. Key exclusion criteria were unstable diabetes with recurrent hypoglycemia, active clinical infection, active autoimmune thyroid disease, history of severe organ dysfunction, malignancy, and history or current or past use of immunosuppressive agents. or details of the recruitment protocol, please see Seelig et al, JCI Insight 2018.

LILACS:
This study recruited hospitalized patients 18 to 85 years age who were admitted with an acute diagnosis of either non-ST elevation MI (NSTEMI) or unstable angina. For details of the recruitment protocol, please see Zhao et al, NEJM Evidence

2021.

Ethics oversight

DILFrequency:
The trial was approved by the Health Research Authority, National Research Ethics Service (14/EE/1057), London, United Kingdom. Informed consent was gained from all participants.

LILACS:
The trial was approved by the UK Greater Manchester Central Research Ethics Committee and the UK Medicines and Healthcare Products Regulatory Agency. Informed consent was gained from all participants.

Note that full information on the approval of the study protocol must also be provided in the manuscript.

Field-specific reporting

Please select the one below that is the best fit for your research. If you are not sure, read the appropriate sections before making your selection.

☒ Life sciences ☐ Behavioural & social sciences ☐ Ecological, evolutionary & environmental sciences

For a reference copy of the document with all sections, see nature.com/documents/nr-reporting-summary-flat.pdf

Life sciences study design

All studies must disclose on these points even when the disclosure is negative.

Sample size

DILFrequency:
Statistical simulations estimated that 36 participants will provide valuable information under scenarios that represent a scientifically plausible and clinically relevant relationship between dose/frequency and the three coprimary end points.

LILACS:
This was an exploratory study that is not designed to formally test a hypothesis. Given that the trial has clinical safety as primary endpoints, a formal power calculation was not relevant.

In the current study, sample size of flow cytometry analysis and scRNAseq of samples from the DILFrequency and LILACS trial were chosen to reflect reasonable minimum numbers required to achieve statistical significance.

Data exclusions

No data was excluded.

Replication

All in vitro experiments were successfully repeated at least twice, often three times, to confirm the findings.
The overall in vivo findings were confirmed in two different clinical trials (DILFrequence and LILACS).

Randomization

DILFrequency:
As a response adaptive trial design, subjects were not randomized. See Seelig et al, JCI Insight 2018 for further details.

LILACS:
Patients were randomly assigned to receive placebo or to one of the two dose groups.

In the current study, randomisation had already been done by the time the samples were analysed by flow cytometry and scRNAseq.

Blinding

DILFrequency:
The trial was a open-label, response-adaptive study. Analysis of flow cytometry data was blinded.

LILACS:
The trial was double-blind. Analysis of flow cytometry data was blinded while analysis of scRNAseq data was unblinded.

In the current study, the investigator conducting the experiment was blinded until the analysis was complete.

Reporting for specific materials, systems and methods

We require information from authors about some types of materials, experimental systems and methods used in many studies. Here, indicate whether each material, system or method listed is relevant to your study. If you are not sure if a list item applies to your research, read the appropriate section before selecting a response.

Materials & experimental systems

n/a	Involved in the study	
[ ]	[x]	Antibodies
[x]	[ ]	Eukaryotic cell lines
[x]	[ ]	Palaeontology and archaeology
[ ]	[x]	Animals and other organisms
[ ]	[x]	Clinical data
[x]	[ ]	Dual use research of concern

Methods

n/a	Involved in the study	
[x]	[ ]	ChIP-seq
[ ]	[x]	Flow cytometry
[x]	[ ]	MRI-based neuroimaging

Antibodies

Antibodies used

Human antibodies:
CD19 (BioLegend, HIB19, 302238)
CD25 (BD Bioscience, M-A251, 555434)
Isotype for above (BD Bioscience, MOPC-21, 555751)
CD25 (BD Bioscience, 2a3, 340907)
Isotype for above (BD Bioscience, X40, 340754)
IL-10 (BioLegend, JES3-9D7, 501404)
TNFα (BioLegend, Mab11, 502912)
CD3 (Abcam, OKT3, ab86883)
Viability Live/Dead (ThermoFisher, L34966)

Murine antibodies:
B220 (Biolegend, RA3-6B2, 103212)
CD25 (Biolegend, PC61, 102030)
Isotype for above (Biolegend, G0114F7, 401910)
Viability Live/Dead (ThermoFisher, L34966)

Validation

Each antibody used in this study is commercially available and have been validated by the respective companies, as well as in previously published studies. All of the antibodies have been referenced in other studies.

Animals and other research organisms

Policy information about studies involving animals; ARRIVE guidelines recommended for reporting animal research, and Sex and Gender in Research

Laboratory animals

All laboratory animals used in this study were C57BL/6 mice 6-12 weeks in age.

Wild animals

No wild animals were used in this study.

Reporting on sex

Samples were collected from both male and female mice. However, we did not analyze the samples on the basis of sex given the small number of mice used in this study.

Field-collected samples

No field collected samples were used in this study.

Ethics oversight

All procedures were carried out in accordance with the United Kingdom Animals (Scientific Procedures) Act of 1986.

Note that full information on the approval of the study protocol must also be provided in the manuscript.

Clinical data

Policy information about clinical studies

All manuscripts should comply with the ICMJEguidelines for publication of clinical research and a completedCONSORT checklist must be included with all submissions.

Clinical trial registration

DILfrequency:
ClinicalTrials.gov NCT02265809

LILACS:
ClinicalTrials.gov NCT03113773

Ethics: Use of splenic tissue: REC reference 15/EE/0152 (East of England - Cambridge South Research Ethics Committee). DILFrequency trial: REC reference 14/EE/1057 (East of England - Cambridge East Research Ethics Committee). LILACS trial: REC reference 17/NW/0012 (North West - Greater Manchester Central Research Ethics Committee). All other experiments involving human tissue are covered under REC reference 12/EE/0446(East of England - Cambridge East Research Ethics Committee).

Study protocol

DILfrequency:

For a complete protocol, please see Truman et al, BMJ Open 2015.

LILACS:
For a complete protocol, please see Zhao et al, BMJ Open 2018.

Data collection

DILfrequency:
Data was collected at the National Institute for Health Research/Wellcome Trust Cambridge Clinical Research Facility, Addenbrooke's Hospital and the University of Cambridge Clinical School over approximately 2 years.

LILACS:
Data was collected at the National Institute for Health Research/Wellcome Trust Cambridge Clinical Research Centre, Cambridge University Hospitals, Cambridge, UK

Outcomes

DILfrequency primary endpoints:
The three coprimary end points were the frequency of Tregs, CD25 expression on Tregs and the frequency of Teffs.

DILfrequency secondary endpoints:
Change in Treg frequency, phenotype and proliferation
Change in Teff frequency, phenotype and proliferation
Change in natural killer (NK) cell frequency, phenotype and proliferation
Change in B lymphocyte cell frequency, phenotype and proliferation
Change in T and NK cell intracellular signalling
Change in full-blood count
Change in plasma/serum levels of IL-2, IL-6, IL-10 and tumour necrosis factor α
Change in metabolic control will be measured by self-monitoring of blood glucose and insulin use; glycated haemoglobin (HbA1c), C-peptide and autoantibody status

LILACS primary endpoints:
The primary outcome will be the safety of IL-2 in patients. This was assessed through:
A review of AEs and SAEs, and concomitant medications.
Changes in safety bloods (electrolytes (sodium, potassium and urea), bone profile (calcium and phosphate), serum creatinine, liver function tests (alanine transaminase, aspartate transaminase, alkaline phosphatase, bilirubin and gamma glutamyl transferase (GT)), thyroid function tests (thyroid stimulating hormone), blood glucose, full blood count and differential, and clotting (prothrombin time and activated partial thromboplastin time).
Twelve-lead ECG and cardiac monitoring changes (arrhythmias, ischaemic changes and QTcB).
Vital observations (blood pressure, heart rate, respiratory rate, peripheral oxygen saturation and temperature).
Echocardiogram changes at baseline and follow-up.

LILACS secondary endpoints:
Change in the mean circulating Treg level measured by fluorescence activated cell sorting (FACS) analysis following treatment with IL-2, over the 5 days of the treatment period.
Change in cardiac biomarker measurements including hs-CRP, troponin I, IL-6 and b-type natriuretic peptide) from analysed blood samples.
Change in lymphocyte subsets measured by FACS analysis.
Pharmacokinetic analysis of IL-2 levels.

Flow Cytometry

Plots

Confirm that:

- [x] The axis labels state the marker and fluorochrome used (e.g. CD4-FITC).
- [x] The axis scales are clearly visible. Include numbers along axes only for bottom left plot of group (a 'group' is an analysis of identical markers).
- [x] All plots are contour plots with outliers or pseudocolor plots.
- [x] A numerical value for number of cells or percentage (with statistics) is provided.

Methodology

Sample preparation

Human peripheral blood B cell CD25 expression
Cells were washed with PBS and blocked for non-specific antibody binding using 1% normal rat serum (ThermoFisher) and 1% human FcR block (Miltenyi Biotec) for 30 minutes. Cells were stained with primary surface antibodies for 30 minutes at room temperature. Cells were washed and stained with a viability stain as per the manufacturer's instructions. Cells were preserved in fixation fluid (produced in-house). Stained leucocytes were processed on a BD LSRFortessa flow cytometer. Flow cytometry files were analysed on FlowJo Version 10 software. Details of all human antibodies used are listed above.

rIL2-treated human blood leucocytes intracellular staining
Prior to primary antibody staining, cells were blocked for non-specific binding with normal rat serum and FcR block as

described above. Extracellullar antibodies were applied as described above. Cells were fixed and stored overnight at 4ºC and permeabilised the following day using an intracellular staining kit as per the manufacturer's instructions (eBioscience). Intracellular stains were applied for 1 hour at room temperature in darkened conditions. Cells were washed and analysed on a flow cytometer. All samples were processed in duplicate along with an unstimulated isotype control. Blood samples from the LILACS study were analysed in a similar fashion to the DILFrequency trial with minor changes outlined in the methods section of the paper.

Human spleen B cell CD25 expression
For experiments involving cell surface expression of the IL2 receptor in B cells from human spleen, cells were stained, and analysed as described above for peripheral blood leucocytes. For co-culture experiments, B cells were stained, fixed, permeabilised and intracellularly stained as per human blood leucocyte samples described above.

Murine spleen B cell CD25 expression
For experiments involving cell surface expression of the IL2 receptor in B cells from murine spleen, cells were stained, and analysed as described above for peripheral blood leucocytes.

For further detailed descriptions of the cell preparation, for example regarding details on stimulation of the cells prior to antibody staining, please see the methods section of the paper.

Instrument	BD LSRFortessa flow cytometer along with BD FACSDiva software for flow cytometry was used for collecting data.
Software	FlowJo version 10
Cell population abundance	In experiments involving B cell isolation, splenocytes and peripheral PBMCs were subsequently resuspended in running buffer. B cells were magnetically negatively isolated (Miltenyi) as per the manufacturer's instruction. If purity was less than 95% by flow cytometry, cells underwent a second round of negative isolation.
Gating strategy	Gating for B cells: Lymphocytes by plotting area of side against area of forward scatter, viability, singlets by plotting height of forward scatter against area of forward scatter, B cells by CD19 staining. Negative and positive populations were distinct. Gating for IL-10: B cells were gated as described above. Borders of IL-10 positive cells were determined by comparing with control samples which did not undergo PMA and ionomycin stimulation.

☒ Tick this box to confirm that a figure exemplifying the gating strategy is provided in the Supplementary Information.
